# Complete response of a metastatic microsatellite-stable gastric cancer after neoadjuvant chemoimmunotherapy: should we still operate? A case report and review of the literature

**DOI:** 10.3389/fonc.2024.1440046

**Published:** 2024-11-20

**Authors:** Hiba Mechahougui, Mickael Chevallay, François Cauchy, Nicolas Chaveau, Giacomo Puppa, Thibaud Koessler, Stefan Monig

**Affiliations:** ^1^ Department of Oncology, Geneva University Hospital, Geneva, Switzerland; ^2^ Department of Visceral Surgery, Geneva University Hospital, Geneva, Switzerland; ^3^ Division of Pathology, Geneva University Hospital, Geneva, Switzerland

**Keywords:** pathological complete response (PCR), gastric cancer, oligometastatic, exceptional responders, conversion surgery, immunotherapy

## Abstract

Gastric cancer often presents at an advanced stage in Western populations due to a lack of screening programs, leading to poor prognoses. Historically, palliative chemotherapy resulted in a median survival of 9.9 months. However, the introduction of the FLOT regimen and immunotherapy has significantly altered treatment outcomes. Oligometastatic gastric cancer, defined as metastasis limited to a single organ or a few sites, has emerged as a distinct subgroup with improved survival when treated with a combination of systemic and local therapies. We present the case of a 54-year-old male patient diagnosed with microsatellite-stable (MSS) oligometastatic gastric adenocarcinoma, including liver and peritoneal metastases, who achieved a complete pathological response following neoadjuvant chemoimmunotherapy with FOLFOX and nivolumab. Despite unfavorable prognostic factors, such as liver involvement and positive peritoneal cytology, the patient responded well to the treatment, allowing curative surgery. Postoperative histology confirmed complete regression of both the primary tumor and metastases, with no recurrence observed at the 1-year follow-up. This case shows the potential of combined chemoimmunotherapy to convert previously inoperable MSS gastric cancer to surgical candidates. Further research is needed to better define patient selection criteria and assess long-term outcomes for these patients.

## Introduction

Due to a lack of a Western screening program, gastric cancer is often discovered at an advanced stage with distant metastases. Historically, patients with gastric cancer were managed with palliative chemotherapy with a median overall survival of 9.9 months (1). The results of the FLOT-3 trial (2) have changed this paradigm. In a group of oligometastatic gastric cancer patients treated with the FLOT regimen (fluorouracil, leucovorin, oxaliplatin, and docetaxel), 36 patients emerged with a good response to systemic treatment and became surgical candidates. These patients with limited metastatic disease exhibited a promising median survival of 31.3 months compared to 15.9 months for patients who did not proceed to surgery. The notion of oligometastatic disease came to light. According to current literature, esophagogastric cancer that has spread to a single organ with ≤3 metastases or to one extraregional lymph node station is classified as an oligometastatic disease. Local treatment for oligometastatic disease has been associated with improved overall survival compared to systemic therapy alone (3). Ongoing efforts are focused on refining the definition of oligometastatic disease through the prospective OligoCare trial (NCT03818503) (4). Besides chemotherapy, immunotherapy has shown encouraging results in palliative metastatic situations. Currently, chemotherapy combined with immunotherapy is the standard first-line treatment for metastatic HER2-negative, programmed cell death ligand 1 (PD-L1)-positive gastric cancers based on the positive results of the CheckMate 649 trial (5). Recently, new players have entered the treatment landscape for this condition, such as anti-claudin 18 therapies (6). Patients who are HER2-positive with PD-L1 >1 can also now benefit from a combination treatment of immunotherapy, trastuzumab, and chemotherapy (7). As a result, the therapeutic landscape for gastric cancer has evolved significantly. However, patients with peritoneal involvement remain underserved by immunotherapy, and deep responses are rare in this population (8). We present the case of a patient with a microsatellite-stable (MSS) oligometastatic gastric adenocarcinoma who despite bad prognosis factors like liver and peritoneal metastases achieved a complete histological response after “neoadjuvant” chemoimmunotherapy.

## Case presentation

**Figure 1:**
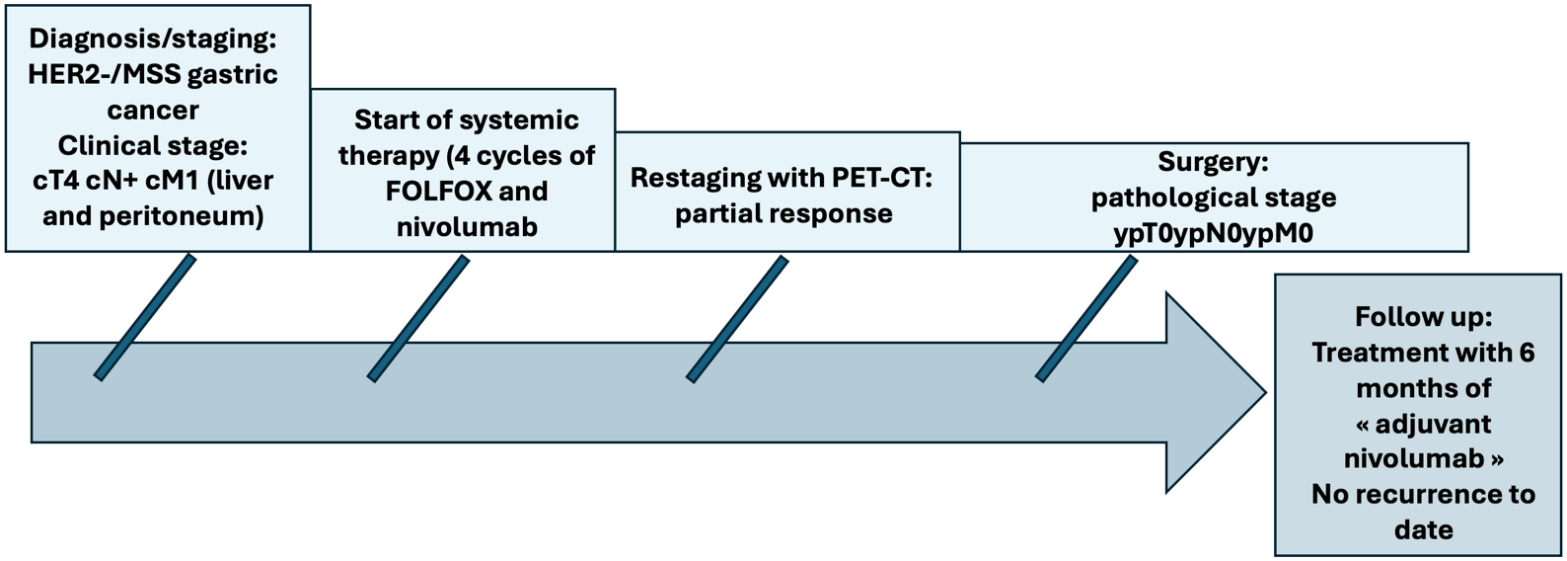
Patient care timeline.

**Figure 2:**
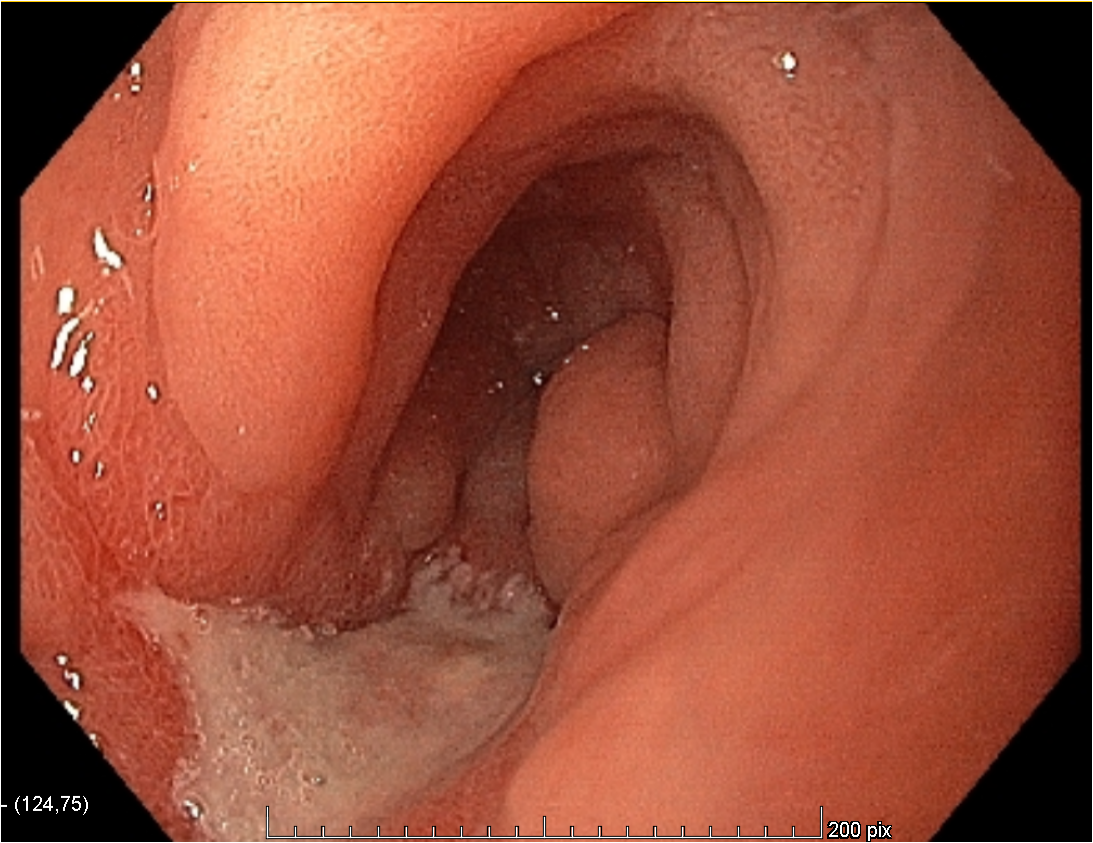
Endoscopic view of the antrum before the neoadjuvant treatment.

**Figure 3:**
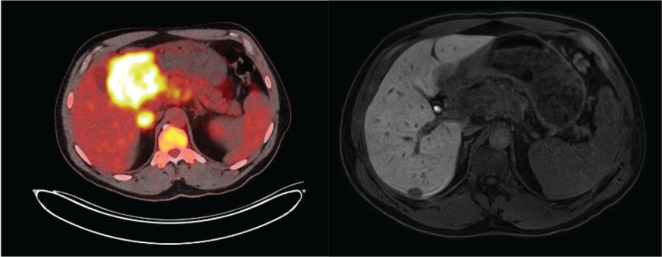
Preliminary staging with PET scan (left) and liver MRI (right). The PET scan shows a clear uptake of the primary tumor with regional lymph nodes. On MRI, a suspicion of liver metastasis is seen just at the junction between segments V and VI.

A 54-year-old patient was addressed to our department with the diagnosis of MSS gastric adenocarcinoma. He had a history of weight loss, dysphagia, and epigastric pain for the last 3 months. He underwent gastroscopy which found an ulcerative lesion in the antrum (**Figures 1**, **2**) and a pangastritis of moderate intensity and activity, with slight atrophy caused by *Helicobacter pylori* infection. The pathological examination confirmed an adenocarcinoma HER2-negative, MSS, and a PD-L1 combined positive score (CPS) of 5. The patient underwent thoraco-abdominal tomography, laparoscopy, and liver MRI, revealing a single suspected liver metastasis between segments V and VII (**Figure 3**). Peritoneal lavage cytology was positive showing suspicious cells. The disease was classified as cT4 cN+cM1 according to the Union for International Cancer Control (UICC 8th edition) (9), and the multidisciplinary tumor board (MDT) proposed a metastatic systemic treatment with chemoimmunotherapy. The patient received four cycles of FOLFOX–nivolumab (leucovorin 400 mg/m², day 1; fluorouracil 400 mg/m², day 1 and 1,200 mg/m², days 1–2; and oxaliplatin 85 mg/m², day 1 and every 2 weeks; nivolumab 240 mg, every 2 weeks).

Restaging with a PET scanner and liver MRI 2 weeks after the fourth cycle showed a partial response (−37%) with downsizing of the gastric lesion, lymph nodes, and liver metastasis (10). An endoscopy examination confirmed macroscopic abnormality, but no biopsy was taken.

**Figure 4:**
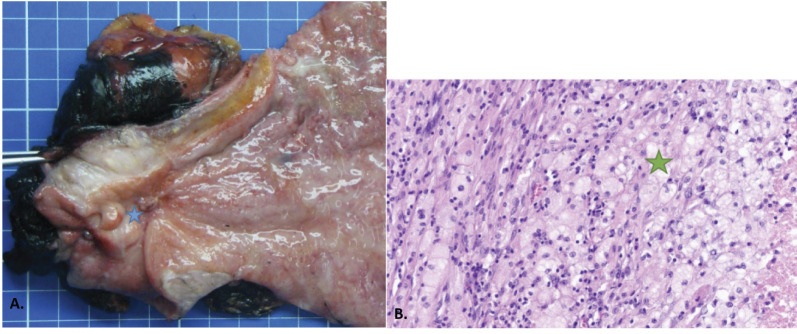
Histopathologic findings. **(A)** Macroscopic appearance with the distal part of the stomach with the tumoral bed (blue star). **(B)** Microscopic appearance with necrosis (orange star) surrounded by a xanthogranulomatous reaction with foamy macrophages (green star), fibrosis, and lymphocytes.

**Figure 5:**
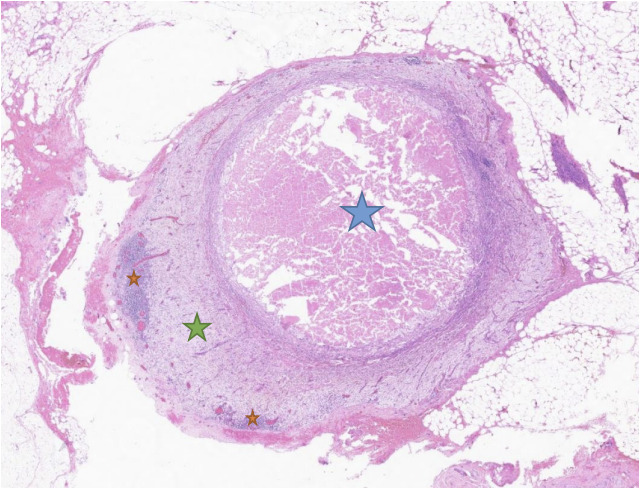
Lymph node with residual parenchyma (orange star) at the periphery, with central necrosis (blue star) and xanthogranulomatous (green star) reaction, which suggests a metastasis regression.

Due to the good partial response, our MDT suggested to conduct a laparoscopic restaging and possible surgery if no carcinomatosis was seen and the primary lesion was resectable. No sign of peritoneal disease was observed, and a hardened lesion at the level of the antrum was palpated but with a possible dissection from the pancreatic plane. In liver segment VI, we found an infracentimetric subcapsular lesion corresponding to the metastasis. We performed a total gastrectomy with a D2 lymphadenectomy and liver metastasectomy with a 1-cm margin. The postoperative course was uneventful, and the patient was discharged on postoperative day 13. On histologic examination of the specimen, a complete pathological response at the primary tumor localization was found with granulomatosis reactions, necrosis, and fibrous inflammatory remnants, conferring a tumor regression grade of 0 (**Figure 4**). Moreover, we found complete pathological regression in 15 out of the 24 examined lymph nodes, as well as in the liver metastasis (**Figure 5**). The final tumor classification was ypT0 ypN0 ypM0 R0. After discussion with the MDT, it was decided to continue with adjuvant nivolumab for a 6-month duration. At the 1-year follow-up, the chest and abdominal scan revealed no evidence of recurrence.

The patient was satisfied with his care and did not experience any significant complications following surgery or adjuvant immunotherapy.

## Discussion

We present the case of an oligometastatic MSS gastric adenocarcinoma with complete pathological response after four cycles of FOLFOX–nivolumab therapy. To our knowledge, this is the first report of such a complete response in a metastatic situation. Despite presenting with two unfavorable criteria (liver metastasis and peritoneal positive cytology) (11), the patient presented a complete regression of the primary and distant tumors. Several case reports of conversion to surgery for metastatic or unresectable gastric cancer have been published (**Table 1**). The distinction in our case lies in the use of a combined chemoimmunotherapy as the initial treatment, with a complete pathological response observed, including in the metastatic sites. The role of immunotherapy in patients carrying microsatellite instability (MSI) is well known in digestive tumors with a median overall survival of 44.8 months for MSI-high patients versus 14.3 months in MSS patients in the CheckMate 649 trial (5). However, our case shows that even patients with MSS status can achieve impressive responses.

**Table 1:** Conversion to surgery case reports for metastatic or unresectable gastric cancer.

Case reports	Age (years)	Molecular profile	Initial staging	Preoperative treatment (number of cycles)	Postoperative staging
Toyota (14)	69	HER2-negative PD-L1 (CPS = 15–20)	cT4aN3bM1 (peritoneal)	First line: S-1 plus oxaliplatin (14) Second line: ramucirumab plus nab-paclitaxel (2) Third line: nivolumab (12)	TRG primary tumor: 1a TRG lymph nodes: 1b
Watanabe (22)	73	HER2-positive	cT4aN2M1 (liver)	First line: capecitabine cisplatin and trastuzumab (12) Second line: Paclitaxel with ramucirumab (25) Third line: nivolumab (31)	ypT3N0M0
Hidaka (23)	69	MSI-high	cT3N2M0	First line: mFOLFOX6 (3) Second line: pembrolizumab (3)	No residual tumor cells
Toyota (13)	75	N/E	uT3N+M1 (peritoneal)	First line: S-1 plus oxaliplatin (2) Second line: paclitaxel plus ramucirumab (7) Third line: nivolumab (23)	ypT2 N0 V0 R0
Matsumoto (24)	68	HER2-negative	cT2N0M1 (liver, lung)	First line: S-1 plus oxaliplatin (6) Second line: ramucirumab plus paclitaxel (6) Third line: nivolumab (20)	No residual tumor cells
Toyota (25)	70	HER2-negative	cT4N`M0	First line: tegafur, gimeracil, and oteracil plus oxaliplatin (3) Second line: paclitaxel plus ramucirumab (7) Third line: nivolumab (24)	ypT1b N1 (1/32) v0 TRG 2b
Kumamoto (26)	69	HER2-negative EBV-negative MSS PD-L1 CPS < 5	cT3N+M1 (lymph node)	First line: S-1 plus oxaliplatin (5) Second line: paclitaxel plus ramucirumab (5) Third line: nivolumab (8)	No residual tumor cells
Lin (27)	69	HER2-negative MSI-high PD-L1 CPS = 40 EBV-positive	T3-4N2M0	First line: paclitaxel arterial infusion and paclitaxel and tislelizumab intravenously (3) Second line: paclitaxel, oxaliplatin, tislelizumab (3)	ypT0N0

HER2, human epidermal growth factor receptor-2; PD-L1 CPS, programmed cell death ligand 1 combined positive score; MSS, microsatellite stability; MSI, high microsatellite instability; EBV, Epstein–Barr virus; FOLFOX, folinic acid, fluorouracil, and oxaliplatin; TRG, tumor regression grade.

The updated CheckMate 649 results showed that in the MSS CPS ≥5 group, the overall response rate was high (60%) with 13 radiologic complete responses, and the outcome of these patients is unknown. Conversely, the presence of a liver metastasis was of poorer prognosis (12). This trend was already noticed in metastatic colon cancer, where the group with liver metastasis had almost no response to immunotherapy, suggesting a colder microenvironment in the liver (10). Regarding the location of the primary tumor, overall survival was higher in gastric cancers than in gastroesophageal junction cancers (15 months versus 13.4 months) (3). This observation is also true for other compounds such as zolbetuximab (antibody against claudin 18.2) in the SPOTLIGHT trial (6).

Peritoneal dissemination has always been the Achilles’ heel of advanced gastric cancer, but systemic immunotherapy seems to be able to control peritoneal carcinomatosis in certain cases (13, 14). In our case, despite a positive cytology, no sign of peritoneal invasion was noted during the surgery. The question of peritoneal penetrance or local control of immunotherapy to avoid peritoneal dissemination is yet to be answered.

Interestingly, our patient presented with *H. pylori* gastritis, which is a well-known carcinogenic agent of gastric cancer. Few studies have been carried out on the subject and patients were not stratified on this parameter in the main trials. Chronic *H. pylori* infection is the leading cause of gastric cancer, accounting for approximately 89% of gastric cancer cases worldwide, and is classified as a class 1 carcinogen by the World Health Organization. Most of the existing data point toward a negative impact of the infection on the immunotherapy response (15).

Our patient therefore presented positive (CPS ≥ 5, gastric location) and negative (MSS status, liver metastasis) predictive factors of response to immunotherapy. In this population of patients who had a pathological complete response after neoadjuvant chemotherapy for advanced gastric cancer, Cho et al. (16) have reported a 5-year disease-free survival (DFS) rate of up to 85%. In the KEYNOTE-585 trial, 12.9% of patients with localized gastric cancer who received chemoimmunotherapy achieved a pathological complete response. However, although the median event-free survival was longer in the pembrolizumab group (44.4 months versus 25.3 months), it did not reach the threshold for statistical significance (17). The second question raised concerns the continuation of immunotherapy following a pathological complete response. The literature on this subject is poor and no prospective study has assessed the issue. Some data exist for metastatic melanomas and show that if a complete response is obtained after systemic treatment, the response seems to be durable with a low probability of long-term recurrence. The place of immunotherapy rechallenge in recurrence is however unclear and deserves further prospective studies (18–20). However, its use earlier in the oncological management could be beneficial for the patient as more toxic treatment can be omitted. By reducing the impact of systemic treatment, patients could be in an enhanced condition to undergo a major surgical procedure.

Despite an improvement in systemic chemotherapy, the surgical principle regarding resection margins is still the same as described in 1980 (21) with a proximal margin of 5 to 8 cm. Patient’s quality of life has been proven to be better after subtotal compared to total gastrectomy. In addition to the benefits of improved survival and disease control, another advantage of immunotherapy treatment could be organ preservation during surgery, allowing for less radical interventions. Finally, if the positive efficacy of immunotherapy continues to be demonstrated, the next challenge will be to identify complete responders. One option, in the case of a complete radiological response, would be to continue with close radiological monitoring, which could potentially avoid the need for extensive surgery. However, for the moment, there is no infallible tool to assess the response. The immunotherapy-induced inflammatory response can appear as a non-response or even an uptake on a PET scan. Additional studies are needed to identify the optimal approach for identifying these patients and providing them with personalized therapeutic strategies.

## Conclusion

Immunotherapy represents a promising and rapidly evolving therapeutic approach for gastric cancer. In oligometastatic disease, a therapeutic approach with combined chemo and immunotherapy achieved local and distant control. Immunotherapy allows to improve outcomes while avoiding intensifying chemotherapy. Further research is needed to better select patients with oligometastatic MSS gastric cancer who will benefit from the triplet combination and could therefore be offered surgery with a chance of a long-lasting cure or possibly even more provocatively cure without surgery.

## Data Availability

The original contributions presented in the study are included in the article/supplementary material. Further inquiries can be directed to the corresponding author.
